# American Medical Association

**Published:** 1880-05-20

**Authors:** 


### *AMERICAN MEDICAL ASSOCIATION.*

This body, composed of representatives of the profession from all parts of the Union, will convene on the first day of June, in the city of New York. A meeting of universal interest and importance is anticipated. In the face of all the criticism that has been made against the Association, it has accomplished good in the past, and is destined, we believe, to exert a great influence and accomplish great good in the future.

The Association of Medical Editors will meet on Monday, the day preceding the opening of the American Medical Association.

The Association of Medical Colleges will meet also to discuss important questions connected with the advancement of medical education.

